# Genomic and functional characterization of *Bacillus subtilis* subsp. *subtilis* INPA R296 with emulsifying activity in cultivation with residual soybean oil

**DOI:** 10.1007/s42770-026-02098-1

**Published:** 2026-10-09

**Authors:** Suziane P. Rodrigues, José C. I. Silva, Nadionara C. Menezes, Rosineide C. de Souza, Aline O. Santos, Igor R. S. Graça, Rogério E. Hanada, Larissa K. Barbosa, Hector H. F. Koolen, Gilvan F. Silva, Luiz A. Oliveira

**Affiliations:** 1 Instituto Nacional de Pesquisas da Amazônia (INPA), Manaus, 69080-971 Brazil; 2 Central de Coleções Microbiológicas (CCM), Universidade do Estado do Amazonas (UEA), Manaus, 69065-001 Brazil; 3 Embrapa Amazônia Ocidental, Manaus, 69010-970 Brazil; 4 Grupo de Pesquisas em Metabolômica e Espectrometria de Massas, Universidade do Estado do Amazonas (UEA), Manaus, 69065-001 Brazil

**Keywords:** Bioconversion, Lipopeptides, Genome mining, UHPLC-HRMS/MS, GNPS

## Abstract

Biosurfactants are amphiphilic microbial metabolites with potential to reduce surface and interfacial tension and to promote emulsification. This study investigated the biosynthetic potential, emulsifying activity, and metabolomic profile of *Bacillus subtilis *subsp.* subtilis* INPA R296 cultivated in media supplemented with residual soybean oil. Phylogenomic analyses supported the taxonomic placement of the strain close to *Bacillus subtilis *subsp.* subtilis* 168^T^. Genome mining predicted 11 biosynthetic gene clusters (BGCs), including NRPS clusters related to surfactin and fengycin biosynthesis. Synteny analyses indicated conservation of these clusters among phylogenetically related *Bacillus* strains. Functional assays showed that culture-medium composition influenced cell growth, emulsification indices, and emulsifying activity. UHPLC-HRMS/MS combined with molecular networking enabled the putative annotation of metabolites belonging to several chemical classes, including cyclic lipopeptides, *N*-acyl amino acids, and oxylipins. The main contribution of this study is the integrated evaluation of genomic biosynthetic potential, emulsifying activity, and putative metabolite annotations in strain INPA R296 cultivated with a low-cost agro-industrial residue. Because no biosurfactant yield, surface/interfacial tension, authentic-standard validation, or structural isolation was performed, the results demonstrate emulsifying potential and the occurrence of putatively annotated amphiphilic metabolites rather than confirmed biosurfactant production. These findings identify INPA R296 as a target for further investigation of amphiphilic metabolites with potential biotechnological applications.

**Supplementary Information:**

The online version contains supplementary material available at https://doi.org/10.1007/s42770-026-02098-1.

## Introduction

The growing concern regarding the environmental impacts associated with the use of petroleum-derived synthetic surfactants has driven the search for biodegradable and less toxic molecules aligned with green chemistry and circular bioeconomy [1, 2]. In this context, microbial biosurfactants stand out due to their biodegradability, low toxicity, and stability under extreme conditions of pH, temperature, and salinity [3, 4]. These amphiphilic molecules reduce surface and interfacial tension, promoting emulsification, dispersion of hydrophobic compounds, and increased bioavailability of organic substrates [4, 5]. Such properties support applications in bioremediation, bioproduct formulation, the pharmaceutical industry, and sustainable agriculture [6–8].

Species of the genus *Bacillus* stand out as biosurfactant producers due to their metabolic versatility and ability to synthesize diverse specialized metabolites [9, 10], as exemplified by *Bacillus subtilis*, which stands out for the production of lipopeptides, including surfactins, fengycins, and iturins, synthesized by nonribosomal peptide synthetases (NRPS) complexes encoded by biosynthetic gene clusters (BGCs) [11–14].

The co-production of these classes of lipopeptides is frequently described in *B. subtilis* and is associated with the plasticity of the specialized metabolism of this species under different conditions [15, 16]. Lipopeptide biosynthesis can be influenced by environmental factors, especially by the availability of hydrophobic carbon sources, such as vegetable oils and agro-industrial residues [17–19]. These substrates may act as precursors of the fatty acid chains involved in lipopeptide biosynthesis, while the use of residual soybean oil represents a strategy for the valorization of agro-industrial residues associated with the reduction of process costs [20]. Despite recent advances, the relationship between the nutritional composition of the medium, expression of biosynthetic gene clusters (BGCs), and structural diversity of the produced metabolites remains poorly understood [21].

Advances in metabolic engineering have explored the regulation of lipopeptide biosynthesis to increase productivity, including the modulation of metabolic fluxes and targeted genetic manipulation [22–24]. Detailed genomic characterization enables the identification of regulatory elements associated with phenotypic differences among strains [25, 26].

Comparative genomics and genome mining are central tools for investigating biosynthetic potential, with platforms such as antiSMASH enabling the identification and comparison of BGCs [27–29]. Integration with Ultra-High Performance Liquid Chromatography coupled to High-Resolution Tandem 

Mass Spectrometry (UHPLC-HRMS/MS) enables comparison of predicted biosynthetic capacity with putatively annotated metabolites, expanding the understanding of specialized metabolism [30–32].

This study aimed to characterize the genomic potential, emulsifying activity, and metabolomic profile of *Bacillus subtilis* subsp. *subtilis* INPA R296 cultivated in media supplemented with residual soybean oil. By integrating phylogenomics, BGC mining, functional emulsification assays, and UHPLC-HRMS/MS-based molecular networking, we sought to determine whether this strain represents a promising source of amphiphilic metabolites and to distinguish genomic biosynthetic potential from experimentally observed emulsifying activity and putative metabolite annotations.

## Materials and methods

### Biological material

The present study was conducted using the strain INPA R296, deposited in the collection of microorganisms of agroforestry interest of the laboratory of ecology and biotechnology of amazonian microorganisms (LEBMAM), at the Instituto Nacional de Pesquisas da Amazônia (INPA), and registered in the National System for the Management of Genetic Heritage and Associated Traditional Knowledge (SISGEN) under registration nº A0DF617. The strain was reactivated by streaking onto Petri plates containing yeast mannitol agar (YMA) medium, followed by incubation at 28 ± 2 °C for 96 h, according to the methodology described by Vincent [33].

### Genomic DNA extraction, sequencing, and assembly

Bacterial cultivation was performed in 10 mL of YM-broth medium under agitation (150 rpm) at 28 °C for 24 h. Cells were collected by centrifugation, and genomic DNA was extracted using the adapted 2% CTAB method [34]. DNA integrity was evaluated by electrophoresis on 0.8% (m/v) agarose gel, and quantification was performed using a Qubit 2.0 fluorometer (Life Technologies) [35].

Sequencing was performed on an Illumina HiSeq platform using paired-end reads (2 × 150 bp), and *de novo* assembly was conducted using SPAdes v. 4.0 with default parameters [36]. Assembly quality was evaluated using the Bacterial and Viral Bioinformatics Resource Center (BV-BRC) platform [37], using tools originally developed at the Pathosystems Resource Integration Center (PATRIC) [38]. The assembly comprised 32 contigs totaling 4,270,493 bp, with an average coverage of 183.57× estimated from contig coverage values. Genome quality estimates indicated 99.9% completeness and 0% contamination, and annotation yielded 4,550 CDSs, of which 884 (19.4%) contained hypothetical annotations.

Genomic data were deposited in the National Center for Biotechnology Information (NCBI) under BioProject PRJNA1427619. The dataset is associated with BioSample SAMN55925958 (INPA R296) and the identification prefix ACZ53Z (Supplementary Fig. S1). The genome assembly is identified by the accession prefix ACZ53Z; a raw-read SRA accession was not available in the current dataset, and the library-preparation details, numbers of raw and quality-filtered reads, and read-filtering criteria were not available in the available genome dataset.

### Phylogenomic analyses and taxonomic delimitation

Phylogenomic analyses and taxonomic identification at the species level were performed by comparing the genome with type strains available in the Type (Strain) Genome Server (TYGS) [39]. Intergenomic distances were estimated using the Genome BLAST Distance Phylogeny (GBDP) method, employing the distance formula d5, and were used for phylogenetic tree reconstruction based on the balanced minimum evolution criterion, as implemented in the software Fast ME v2.1.6.1 [40], with topological optimization by Subtree Pruning and Regrafting (SPR) and statistical support evaluated by pseudo-bootstrap analysis with 1000 replicates. The trees were rooted using the midpoint method.

Taxonomic delimitation was evaluated based on digital DNA–DNA hybridization (dDDH) values, calculated in TYGS using formula d4, adopting the 70% species boundary, with values ≥ 70% supporting assignment within the same species. Average nucleotide identity (ANI) was estimated using the OrthoANI [41] and FastANI [42] software packages, considering a cutoff value of 96.7%. Complementary ANI analyses (ANIb and ANIm) were performed using the JSpeciesWS platform [43–45], while average amino acid identity (AAI) was estimated using the Enveomics platform [46], adopting the 95% species boundary, with values ≥ 95% supporting assignment within the same species as an auxiliary criterion [47]. An independent phylogenomic analysis was conducted using the AutoMLST (Automated Multi-Locus Species Tree) platform [48], based on the concatenation of conserved single-copy genes (Supplementary Table S3).

Heatmaps based on ANI and AAI matrices were generated using the ChiPlot platform (https://www.chiplot.online/) for visualization of genomic similarity patterns. The valid nomenclature of related species was verified based on the List of Prokaryotic names with Standing in Nomenclature (LPSN) [49].

### Genome mining of the biosynthetic potential for specialized metabolites

The genome was analyzed using the antiSMASH v8 platform for the prediction of biosynthetic gene clusters (BGCs) [28], and functional annotation and identification of protein domains associated with the BGCs were performed using the InterPro [50], PKS/NRPS Analysis [51], and SMART [52] databases. The antiSMASH output corresponded to version 8.0.4 and used the assembled FASTA file (Strain_INPA_R296.fasta) as input.

BGC delimitation was based on the identification of core biosynthetic genes and accessory genes related to the production of specialized metabolites. The structural conservation and synteny of the BGCs, in comparison with genomes of species of the genus *Bacillus*, were evaluated using the Clinker pipeline in conjunction with the Clustermap.js tool [53].

### Cell growth and emulsifying activity

The pre-inoculum was washed with sterile saline solution before preparation of the bacterial suspension. The bacterial suspension was adjusted to turbidity equivalent to the 0.5 McFarland standard (10⁸ CFU·mL⁻¹) and inoculated into 100 mL of the following liquid media supplemented with residual soybean oil (1% v/v): YM, INPA, yeast salts (SY), and yeast extract (YE), at pH 7.0 (Table 1). Water supplemented with residual soybean oil was also inoculated and used as a control. Media without residual soybean oil were not evaluated. The residual soybean oil was obtained after domestic use. It was selected as a readily available, low-cost residual feedstock for waste valorization and circular-bioeconomy applications; however, its physicochemical composition was not characterized and substrate consumption was not measured. The carbon contribution of the complex media components, including yeast extract and mannitol where applicable, is provided in Table 1. Cultures were incubated at 28 ± 2 °C under agitation at 150 rpm, using three independent biological replicates for each cultivation condition. Samples were collected at regular intervals up to 144 h of incubation, and OD₆₀₀ was measured for each replicate at each sampling time using a Bio-Rad iMark™ microplate spectrophotometer. Because the study did not quantify the amount of biosurfactant recovered, the functional results are reported as emulsifying activity and emulsifying potential rather than biosurfactant production yield.

**Table 1:** Composition of the culture media used to evaluate bacterial growth and emulsifying activity

Growth Medium	Composition (g.L^− 1^)
INPA	0.4 – MgSO₄·7 H₂O; 0.02 – Ca(CH₃COO)₂·H₂O; 1.5 – KH₂PO₄; 0.5 – K₂HPO₄; 2.5 – (NH₄)₂SO₄·7 H₂O; 0.05 – FeCl₃·6 H₂O; 0.05 – FeSO₄·7 H₂O; 0.01 – ZnSO₄·7 H₂O; 0.01 – CuSO₄·5 H₂O; 0.01 – MnSO₄·7 H₂O; 0.01 – Na₄SeO₃.
YE	0.5 – Yeast extract.
SY	0.5 – Yeast extract; 2.0 – KH₂PO₄; 0.2 – MgSO₄·7 H₂O; 0.1 – NaCl.
YM	10.0 – Mannitol; 0.5 – Yeast extract; 2.0 – KH₂PO₄; 0.2 – MgSO₄·7 H₂O; 0.1 – NaCl.

#### Determination of emulsifying activity

Emulsifying activity was evaluated using the whole culture broth, including cells and extracellular material; the broth was not centrifuged before the assay. Emulsifying activity was evaluated using three independent biological replicates for each cultivation condition at 24 and 48 h of cultivation. Each replicate consisted of an independent culture broth. The assay blank was the corresponding uninoculated medium supplemented with residual soybean oil; for the water-plus-oil condition, water supplemented with residual soybean oil without inoculum was used as the blank. For the O/W response, the values used for Fig. 8D corresponded to the mean absorbance measured at 610 nm. Residual soybean oil was added at a 1:1 (v/v) ratio, and the mixture was homogenized in a vortex mixer (Kasvi) at 700 rpm for 2 min. After 1, 24, and 48 h of rest, the 24 and 48 h measurements were selected for presentation as early and intermediate emulsion-stability endpoints; they represented early and intermediate emulsion-stability endpoints rather than the complete growth period. Oil-in-water (O/W) emulsifying activity was determined by spectrophotometry at 610 nm, whereas the water-in-oil (W/O) emulsification index was determined from the ratio between the height of the formed emulsion and the total height of the mixture. The aqueous phase was not used as the denominator in the alternative layer-based calculation [54, 55]. The distinction between these measurements is relevant because O/W activity is based on absorbance, whereas EI% and W/O-related values are based on layer heights; therefore, these parameters should not be directly compared without normalization.

Oil-in-water emulsifying activity (EA_O/W_) was represented by the sample absorbance at 610 nm multiplied by D, where D = 1 because the whole culture broth was analyzed without dilution (Eq. 1):

1 $$\:E{A}_{o/w}=(Ab{s}_{sample}-Ab{s}_{blank})\cdot\:D$$

The emulsification index (EI%) was determined by the ratio between the height of the formed emulsion and the total height, multiplied by 100 (Eq. 2):

2 $$\:EI{\%}=\left(\frac{{h}_{emulsion}}{{h}_{total}}\right).100$$

Water-in-oil emulsifying activity (EA_W/O_) was calculated as the difference between the emulsification indices of the sample and the blank, multiplied by D, where D = 1 because the whole culture broth was analyzed without dilution (Eq. 3):

3 $$\:E{A}_{w/o}=({EI}_{sample}-{EI}_{blank})\cdot\:D$$

Because the amount of biosurfactant or other emulsifying material was not quantified, the EP_W/O_ parameter was interpreted only as a time-normalized emulsification response, not as a production yield. EP_W/O_ was calculated as EA_W/O_ divided by the corresponding incubation time and is not reported as a rate of biosurfactant production.

$$\:E{P}_{w/o}=\frac{EA}{t}$$

In this study, EU denotes an operational emulsifying-unit response calculated under the stated assay conditions; it was not interpreted as a mass-normalized biosurfactant yield.

#### UHPLC-HRMS/MS analysis

Cultivations were performed using 100 mL of liquid media supplemented with residual soybean oil at 1% (v/v). YM, INPA, SY and YE media were evaluated, together with a control consisting only of water and residual soybean oil. The pH of the media was adjusted to 7.0 using 10 M NaOH and 10 M HCl solutions. Bacterial inoculation was performed using a cell suspension standardized to 0.5 McFarland (10⁸ CFU·mL⁻¹), obtained by spectrophotometer reading at 600 nm. Cultures were incubated for 196 h at 28 ± 2 °C under agitation at 150 rpm. The functional assays and metabolomic sampling used different cultivation durations because they addressed distinct experimental objectives: functional measurements followed growth and emulsification over time, whereas the 196 h cultures were used to obtain material for comparative metabolomic profiling. Direct temporal equivalence between these datasets was therefore not assumed.

After incubation, 50 mL of each culture were collected for extract preparation. Samples were subjected to ultrasonic bath treatment followed by lyophilization using an Alpha 1–2 LSCbasic system (Christ). The lyophilized extracts were resuspended in 30 mL of methanol, sonicated for 15 min, and vacuum-filtered using a porous plate funnel coupled to a 47 mm filter paper membrane. The solvent was removed using a rotary evaporator at 40 °C and 60 rpm.

Samples (0.9 mg of crude extract) were resuspended in 1 mL of LC-MS-grade methanol, sonicated for 5 min, centrifuged at 10,000 rpm for 10 min, and filtered through a PVDF membrane (0.22 μm). Analyses were performed using a Vanquish Flex UHPLC system (Thermo Scientific) coupled to an Orbitrap Exploris™ 240 mass spectrometer equipped with a HESI source, operating in positive and negative ionization modes. Acquisition was conducted in the *m/z* range of 150–2000, with a resolution of 120,000 for full scan and 15,000 for MS/MS.

Chromatographic separation was performed using a Hypersil GOLD column (50 × 3.0 mm; 1.9 μm), maintained at 40 °C, with a flow rate of 0.3 mL·min⁻¹ and an injection volume of 2 µL. In positive mode, the mobile phase consisted of water and methanol containing 0.1% formic acid; in negative mode, water and methanol containing 5 mM ammonium formate. The gradient started at 10% phase B, reaching 50% at 5 min and 100% between 5.0 and 12.5 min, followed by a return to the initial conditions and re-equilibration until 15 min.

The MS/MS experiments were conducted in DDA mode, selecting the five most intense precursor ions per cycle, with an isolation window of 1.5 *m/z* and HCD fragmentation using normalized collision energies of 30, 50, and 70%. Ions with a minimum intensity of 5,000 were considered, applying a dynamic exclusion of 5 s and a mass tolerance of 5 ppm [56].

Metabolomic analyses were performed using five independent biological replicates for each cultivation condition: YE, SY, YM, INPA, and water supplemented with residual soybean oil as the control. Six methanol blanks were included during data acquisition. Each biological replicate corresponded to a single LC-MS file in the GNPS metadata. For each experimental condition, the acquisition sequence consisted of the methanol blank, the culture-medium control, and subsequently the corresponding samples. The feature-intensity matrix generated by MZmine was used to construct the feature-based molecular network. Accordingly, the results are presented as relative feature abundances and putative metabolite annotations rather than absolute metabolite concentrations.

#### Statistical analysis

Functional assays were conducted using three independent biological replicates per cultivation condition, and results are presented as mean ± standard deviation. The data were analyzed descriptively because the available functional dataset was not structured for a full factorial inferential analysis across culture media and incubation times; therefore, no hypothesis tests, post hoc comparisons, or significance lettering were applied. Metabolomic data were obtained from five independent biological replicates per condition and are reported as relative feature abundances and putative metabolite annotations rather than absolute concentrations.

#### Feature-based molecular networking (FBMN)

UHPLC-HRMS/MS data were processed using the MZmine v4 software and analyzed by feature-based molecular networking (FBMN) on the GNPS2 platform. Processing included signal detection at the MS1 and MS/MS levels, chromatographic construction and deconvolution, alignment among samples, and removal of redundant signals and signals derived from analytical blanks. Additionally, the ion identity networking approach was applied for grouping different ionic forms derived from the same metabolite, as well as putative lipid annotations based on spectral data. The MS/MS spectra, the quantitative feature matrix, and experimental metadata were exported to GNPS2 for molecular network construction.

The networks were constructed based on the similarity between experimental spectra and GNPS spectral libraries using the cosine coefficient as the similarity metric. Only spectra with a cosine score ≥ 0.6 and a minimum of five matched fragments were considered. Mass tolerance was set at 0.02 Da for precursor and fragment ions. The search for spectral analogs was disabled. All compound assignments based on GNPS spectral matching are putative annotations consistent with MSI level 2 criteria; no authentic standards, targeted MS/MS validation, or structural isolation was performed.

The molecular networks generated by FBMN analysis are publicly available on the GNPS2 platform at the following links: https://gnps2.org/status?task=65e54cee17934a72a4923e157b1259f9 (Negative mode) and https://gnps2.org/status?task=11a3c2831c68439dba114fcb1a1dab73 (positive mode).

## Results and discussion

### Genomic characterization and phylogenomic positioning

The genome of strain INPA R296 comprised 4,270,493 bp distributed across 32 contigs, with an N50 of 639,189 bp and an L50 of 3. The average coverage estimated from contig coverage values was 183.57×. Genome quality estimates indicated 99.9% completeness and 0% contamination. Functional annotation identified 4,550 CDSs, of which 884 (19.4%) contained hypothetical annotations. These objective metrics are reported here without referring to the assembly as high quality in the absence of a formal quality standard.

**Fig. 1:**
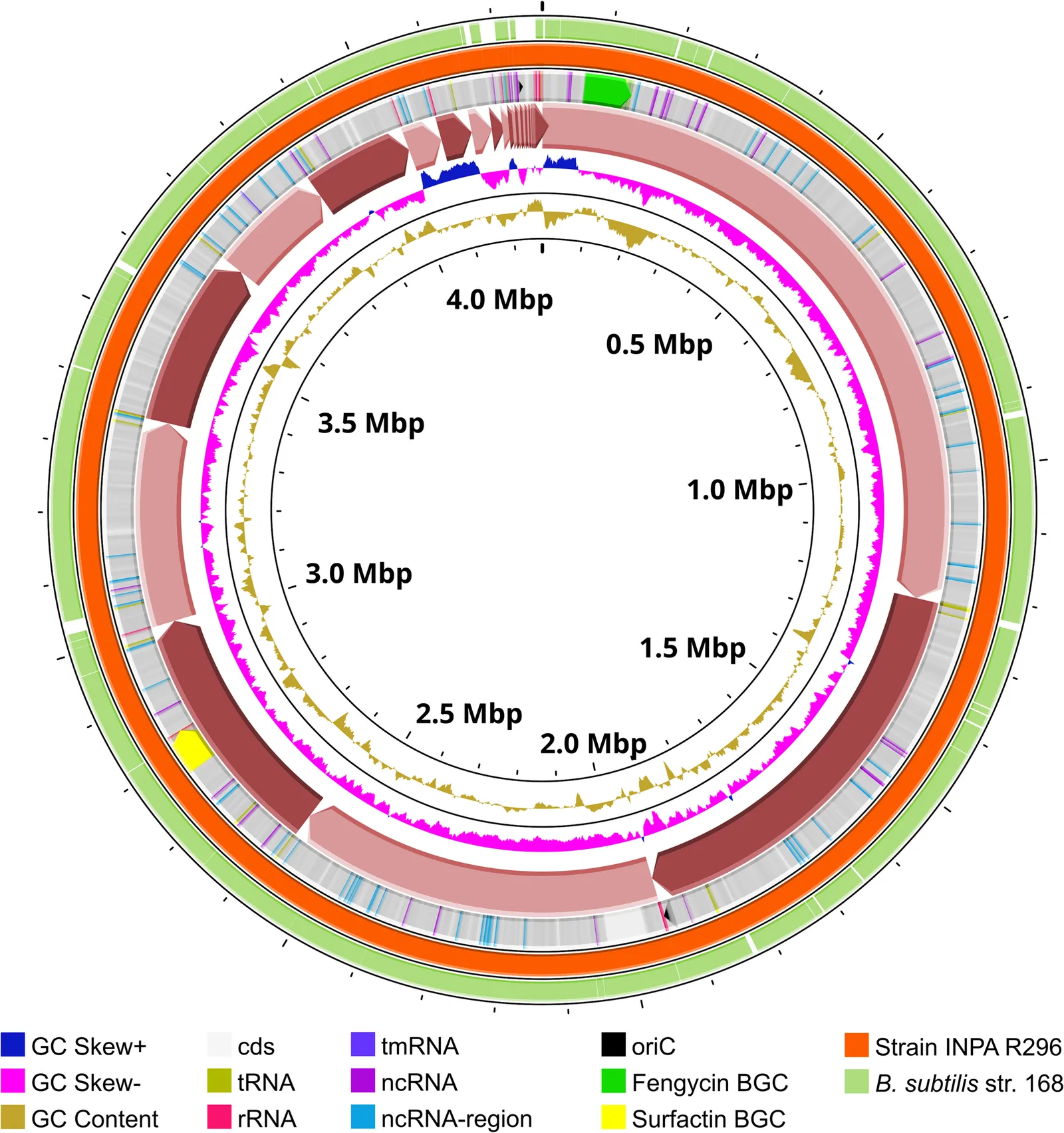
Circular genomic map of *B. subtilis* subsp. *subtilis* INPA R296. The rings represent, from the outermost to the innermost, genomic similarity based on BLAST against *B. subtilis* subsp. *subtilis* 168ᵀ, protein-coding genes (CDSs) on both strands, ribosomal RNAs and transporters, GC content, and GC skew. NRPS-type biosynthetic clusters associated with fengycin and surfactin biosynthesis are highlighted in contigs 1 and 4, respectively

The circular genomic map generated on the Proksee platform revealed a homogeneous distribution of protein-coding genes, as well as conserved patterns of GC content and GC skew throughout the genomic assembly (Fig. 1). Structural comparison based on BLAST with the reference genome *Bacillus subtilis* subsp. *subtilis* 168ᵀ revealed high genomic similarity and collinearity between the strains, characterized by conservation of genomic organization and distribution of the main annotated elements.

**Fig. 2:**
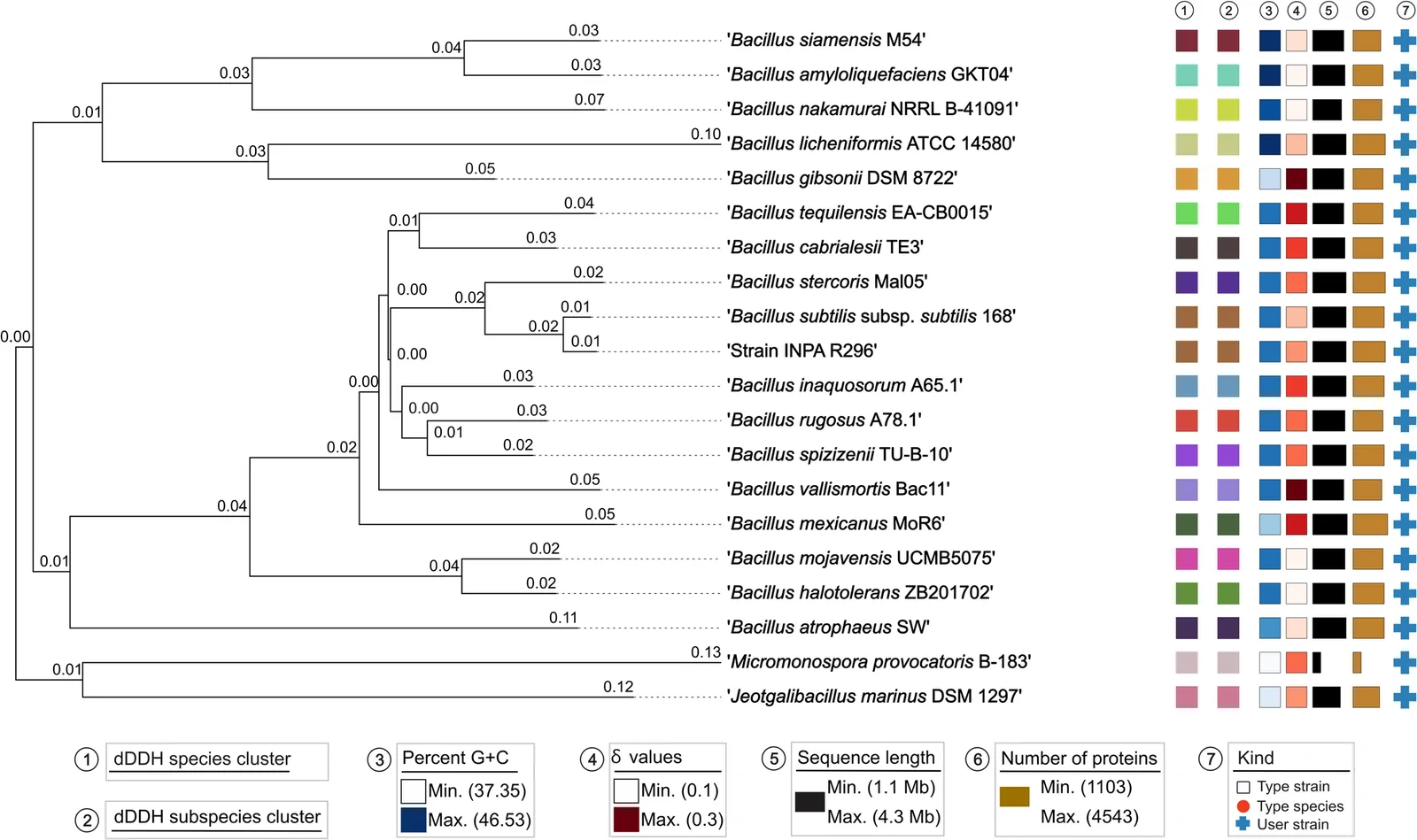
Phylogenomic reconstruction based on whole genomes obtained by the TYGS server, showing the evolutionary relationships between strain INPA R296 and type strains of the genus *Bacillus*. The tree was inferred using the FastME v2.1.6.1 algorithm from GBDP distances calculated using formula d5. Branch lengths represent the estimated genomic distances, whereas the values indicated at the nodes correspond to GBDP pseudo-bootstrap values (> 60%) obtained with 1000 replicates, showing an average support of 92.5%. The tree was rooted using the midpoint method

Whole-genome-based phylogenomic reconstruction positioned strain INPA R296 close to the type strain *B. subtilis* subsp. *subtilis* 168ᵀ, forming an adjacent branch in the tree inferred by the TYGS server (Fig. 2). Related species, including *B. stercoris* Mal05ᵀ, *B. tequilensis* EA-CB0015ᵀ, *B. vallismortis* Bac11ᵀ, *B. mojavensis* UCBM5075ᵀ, and *B. licheniformis* ATCC 14580ᵀ, were distributed into independent clades, evidencing the phylogenomic separation among the analyzed taxa.

**Fig. 3:**
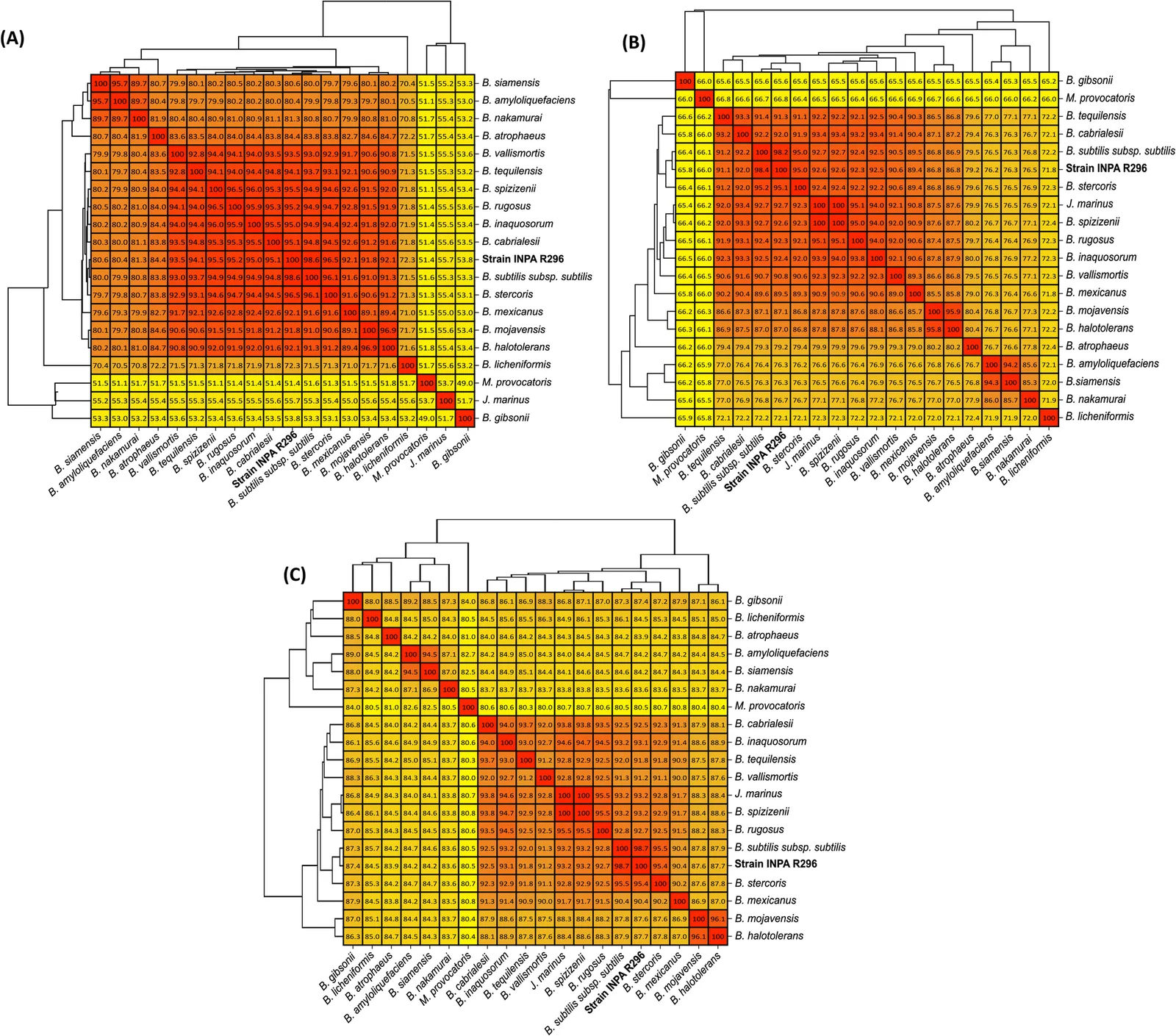
Genomic similarity matrices among strains of the genus *Bacillus* generated using Chiplot. (A) AAI matrix; (B) ANIb matrix; and (C) ANIm matrix. Pairwise genomic similarity values are displayed as a heatmap, allowing the evaluation of nucleotide identity levels among the analyzed strains and assisting in taxonomic delimitation at the species level

Genomic comparisons reinforced this taxonomic positioning. The dDDH value between INPA R296 and *B. subtilis* subsp. *subtilis* 168ᵀ was 88.8% (confidence interval: 86.3–90.8%), above the 70% threshold adopted for bacterial species delimitation, whereas the difference in G + C content was only 0.18% (Supplementary Tables S1 and S2). Similarly, similarity matrices based on AAI, ANIb, and ANIm demonstrated consistent clustering between INPA R296 and *B. subtilis* subsp. *subtilis* 168ᵀ, with values higher than 98% across all evaluated approaches (Fig. 3, **S**upplementary Table S3). In contrast, more distantly related species of the genus exhibited lower levels of genomic similarity, forming separate clusters in the heatmaps. The concordance among metrics based on nucleotide and protein identity indicates that the taxonomic positioning of the strain is consistent across these approaches.

**Fig. 4:**
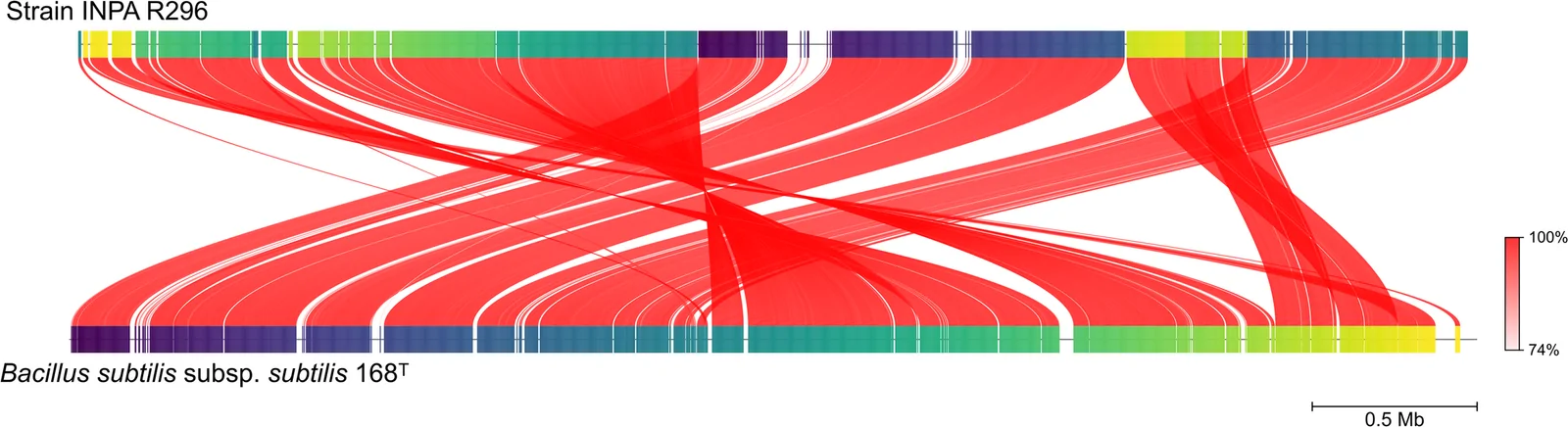
Average nucleotide identity (ANI) analysis between *B. subtilis* subsp. *subtilis* INPA R296 and *B. subtilis* subsp. *subtilis* 168ᵀ, performed using the FastANI tool on the Proksee platform. The ANI value was 98.52%. Each red line segment represents reciprocal mapping between the query genome and the reference genome

Complementing these analyses, synteny evaluation based on FastANI revealed high collinearity between the genomes of INPA R296 and *B. subtilis* subsp. *subtilis* 168ᵀ, evidenced by the presence of extensive conserved blocks and maintenance of gene order throughout the chromosome (Fig. 4). The few observed interruptions likely correspond to variable regions or strain-specific genomic differences. Together, phylogenomic, genomic similarity, and synteny data consistently support the classification of strain INPA R296 as *B. subtilis* subsp. *subtilis*.

### Genome mining of BGCs

The genome of strain INPA R296 presented 11 BGCs associated with different classes of specialized metabolites, including NRPS, polyketides (PKS), hybrid NRPS-PKS clusters, ribosomally synthesized and post-translationally modified peptides (RiPPs), terpenes, and metallophores (Table 2). Seven clusters showed high similarity to previously characterized BGCs in the MIBiG database, indicating biosynthetic potential consistent with the production of bioactive metabolites commonly associated with the genus *Bacillus*. The surfactin- and fengycin-related clusters were selected for detailed analysis because of their relevance to the study objective and their high similarity to characterized reference clusters; this selection does not imply that the remaining BGCs are inactive or biologically unimportant.

**Table 2:** Annotated BGCs in the genome of strain INPA R296

Region	Type	From	To	Similarity confidence	Most similar known cluster	MIBiG accession(s)
1.1	NRPS, betalactone	66,409	144,117	High	fengycin	BGC0001095
1.2	T3PKS	327,588	368,685	Low	1-carbapen-2-em-3-carboxylic acid	BGC0001847
1.3	terpene-precursor	550,871	571,494	-	-	-
2.1	CDPS	60,739	81,125	High	pulcherriminic acid	BGC0002103
2.2	sactipeptide	300,110	321,387	High	subtilosin A	BGC0000602
2.3	other	324,374	365,792	High	bacilysin	BGC0001184
2.4	RRE-containing	560,499	580,768	-	-	-
3.1	transAT-PKS, NRPS-like, NRPS, T3PKS, PKS-like	10,822	125,577	High	bacillaene	BGC0001089
4.1	NRPS	188,648	254,036	High	surfactin	BGC0000433
5.1	terpene	134,096	154,899	-	-	-
8.1	NRP-metallophore, NRPS	82,173	133,95	High	bacillibactin	BGC0000309

#### Characterization of the NRPS cluster related to fengycin biosynthesis

An NRPS cluster associated with beta-lactone identified in *B. subtilis* subsp. *subtilis* INPA R296 showed high similarity to the fengycin BGC deposited in MIBiG. Functional annotation identified enzymes related to lipid precursor and acyl-CoA metabolism, including UTP–glucose-1-phosphate uridylyltransferase, methylcrotonyl-CoA carboxylase, methylglutaconyl-CoA hydratase, and hydroxymethylglutaryl-CoA lyase, potentially involved in supplying metabolic units for lipopeptide biosynthesis (Supplementary Table S4) [57].

**Fig. 5:**
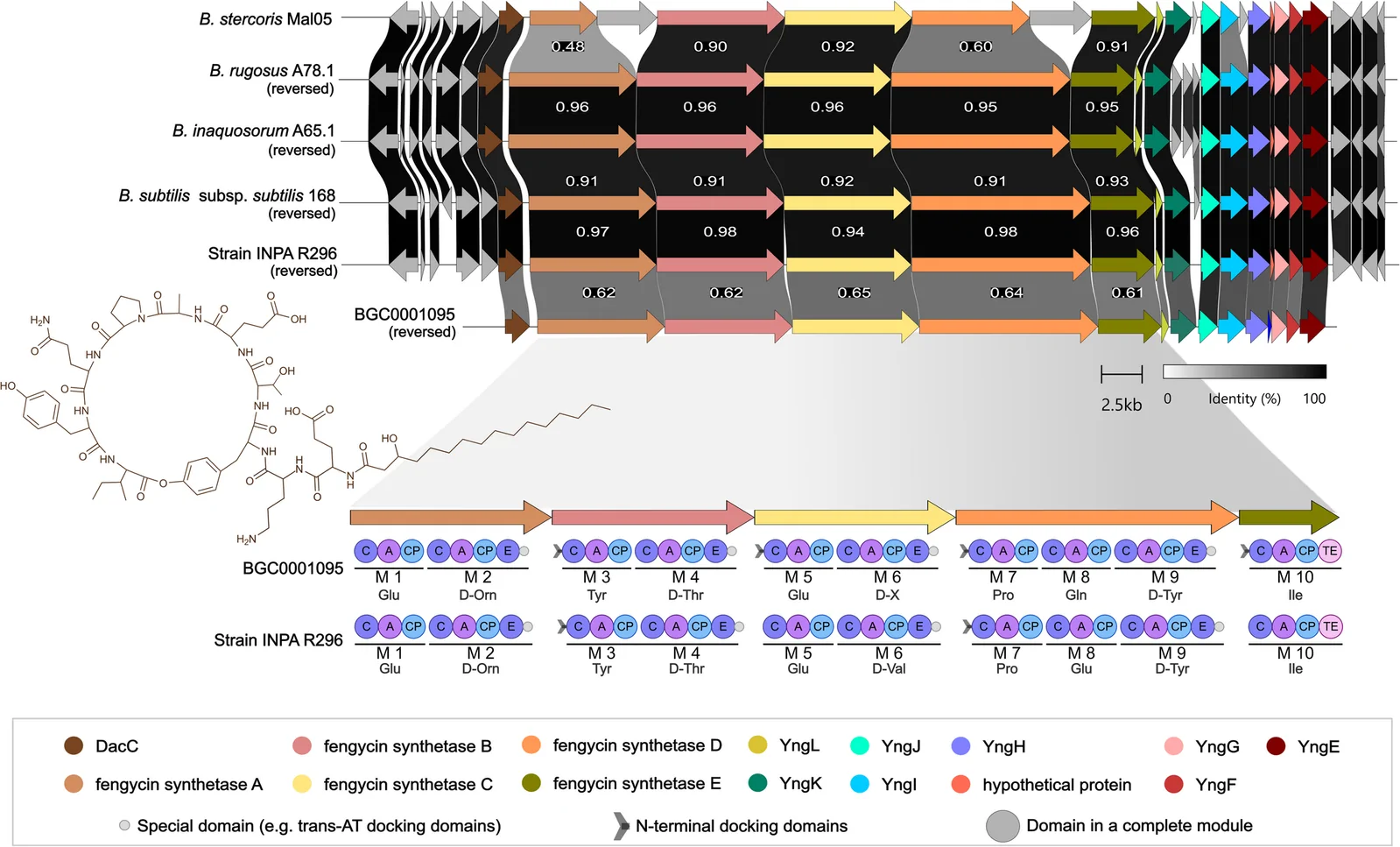
Synteny analysis of the fengycin BGC in phylogenetically related strains compared with the reference cluster BGC0001095 deposited in MIBiG. Arrows represent coding genes and the direction of transcription. Shaded regions and numerical values indicate sequence identity among orthologous genes. The modular organization of the NRPS system includes adenylation (A), condensation (C), peptidyl carrier protein (PCP), and epimerization (E) domains. A representative chemical structure of fengycin is shown on the left side of the figure

Comparative synteny analysis demonstrated high structural conservation relative to homologous fengycin BGCs from *B. stercoris* Mal05, *B. rugosus* A78.1, *B. inaquosorum* A65.1, *B. subtilis* subsp. *subtilis* 168^T^, and the reference cluster BGC0001095 (Fig. 5). The core genes (*fenA*–*fenE*) maintained conserved order and orientation, with high sequence identity among orthologs. Accessory genes related to regulation, modification, and transport were also conserved, whereas variations were mainly restricted to flanking regions. Modular differences among NRPS systems may indicate variation in biosynthetic specificity, although spectrometric validation is still required. The presence of this BGC is therefore interpreted as genomic biosynthetic potential and does not establish fengycin production under the conditions evaluated.

#### Characterization of the NRPS cluster related to surfactin biosynthesis

An NRPS-type BGC identified in *B. subtilis* subsp. *subtilis* INPA R296 showed high similarity to the surfactin cluster deposited in MIBiG. Functional annotation identified accessory enzymes potentially involved in product activation and release, including 4′-phosphopantetheine transferase and thioesterase, in addition to genes associated with transport (ABC and MFS systems) and transcriptional regulation (Supplementary Table S5). This functional profile is consistent with biosynthetic systems involved in amphiphilic lipopeptide production [58–61]. 

**Fig. 6:**
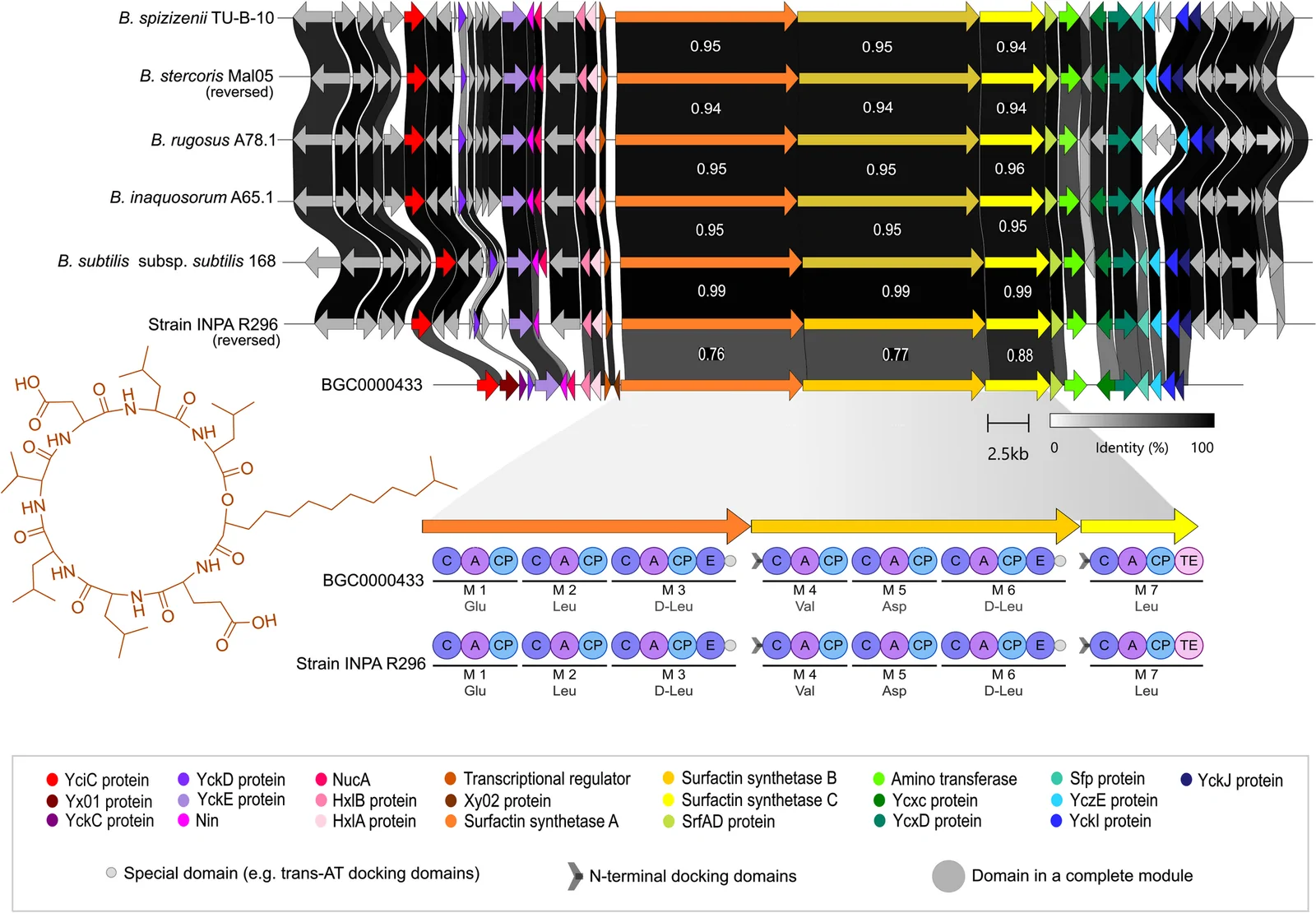
Synteny analysis of the surfactin BGC in *Bacillus* strains compared with the reference cluster BGC0000433 deposited in MIBiG. Arrows represent coding genes and the direction of transcription. Shaded regions and numerical values indicate sequence identity among orthologous genes. The modular organization of the NRPS system includes condensation (C), adenylation (A), peptidyl carrier protein (PCP), epimerization (E), and thioesterase (TE) domains. A representative chemical structure of surfactin is shown on the left side of the figure

Comparative synteny analysis revealed high structural conservation relative to homologous surfactin BGCs from *B. spizizenii* TU-B-10, *B. stercoris* Mal05, *B. inaquosorum* A65.1, *B. rugosus* A78.1, *B. subtilis* subsp. *subtilis* 168ᵀ, and the reference cluster BGC0000433 (Fig. 6). The compared strains maintained high gene collinearity and conserved organization of NRPS structural genes. Although sequence-level differences and modular variations were observed, the overall biosynthetic architecture remained conserved, suggesting possible variation in lipopeptide biosynthetic specificity.

The identified BGCs are consistent with the metabolic potential described for the *B. subtilis* complex, characterized by conserved NRPS pathways associated with specialized metabolites [30, 61–63]. Metabolites such as surfactin and fengycin have been linked to motility, biofilm formation, and microbial interactions [57, 63]. Together with previous reports of simultaneous surfactin and fengycin production in *B. subtilis* cultures [64], these findings support the potential of strain INPA R296 to encode cyclic lipopeptide biosynthetic pathways. They do not, however, demonstrate that individual BGCs were expressed or that they generated specific metabolites detected in this study.

### Evaluation of emulsifying potential

**Fig. 7:**
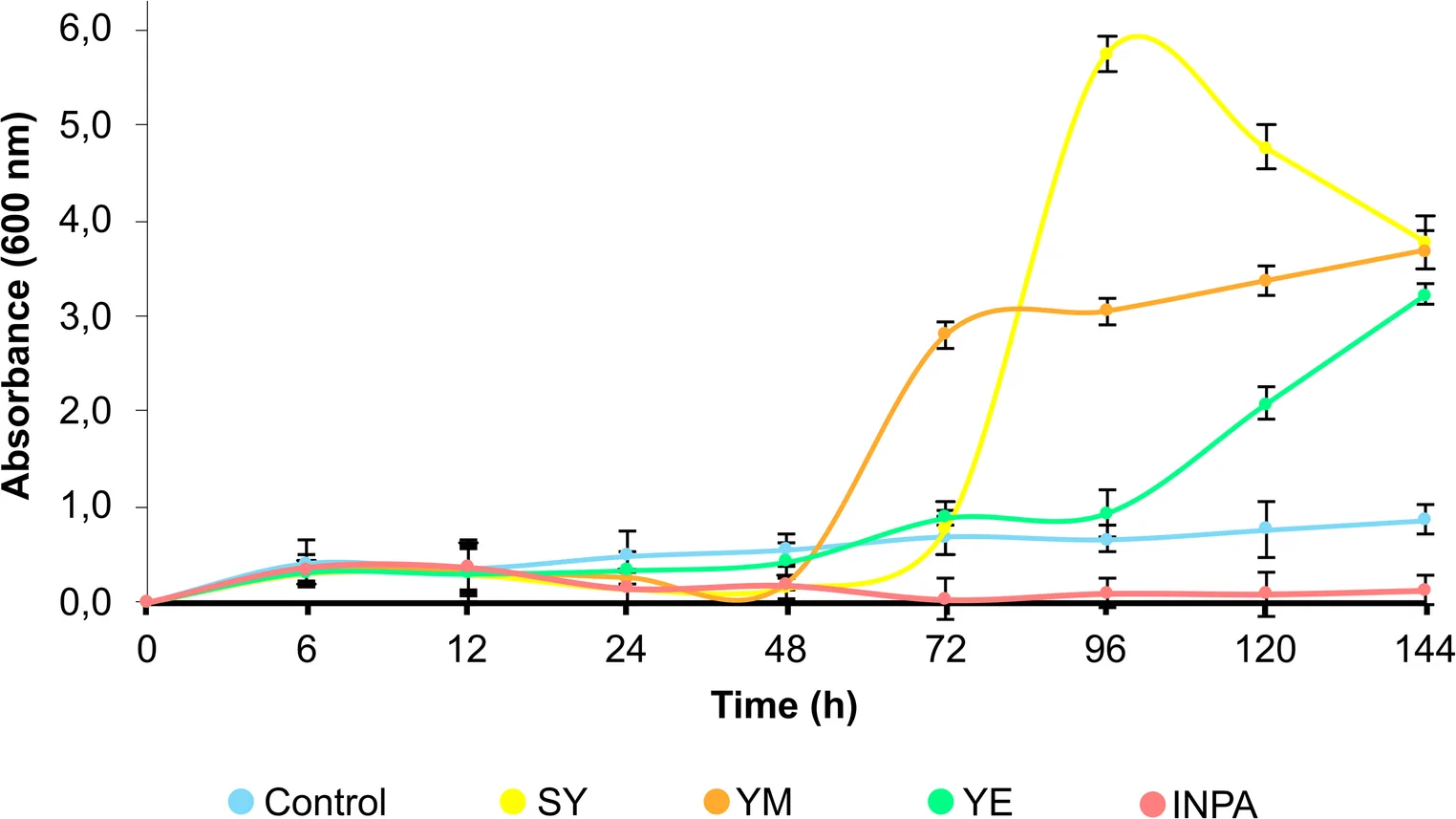
Cell growth expressed as OD₆₀₀

Considering the presence of NRPS BGCs associated with lipopeptide biosynthesis in the genome of strain INPA R296, emulsifying activity was evaluated in different culture media throughout the experimental period. Bacterial growth varied among the evaluated conditions (Fig. 7). The highest OD₆₀₀ values were observed in YM and SY media, which reached peak growth at 72 h and 96 h, respectively, suggesting that medium composition, including the presence or absence of mannitol, may influence growth dynamics under the evaluated conditions [65, 66].

The conditions containing YE and Control exhibited similar initial growth; however, the medium supplemented with YE showed a progressive increase in biomass throughout incubation, possibly due to the availability of complex nitrogen sources favoring the maintenance of metabolic activity and cellular proliferation [67].

In contrast, the INPA medium exhibited reduced growth during cultivation, possibly as a result of its mineral composition and the lower availability of complex organic nutrients [68].

**Fig. 8:**
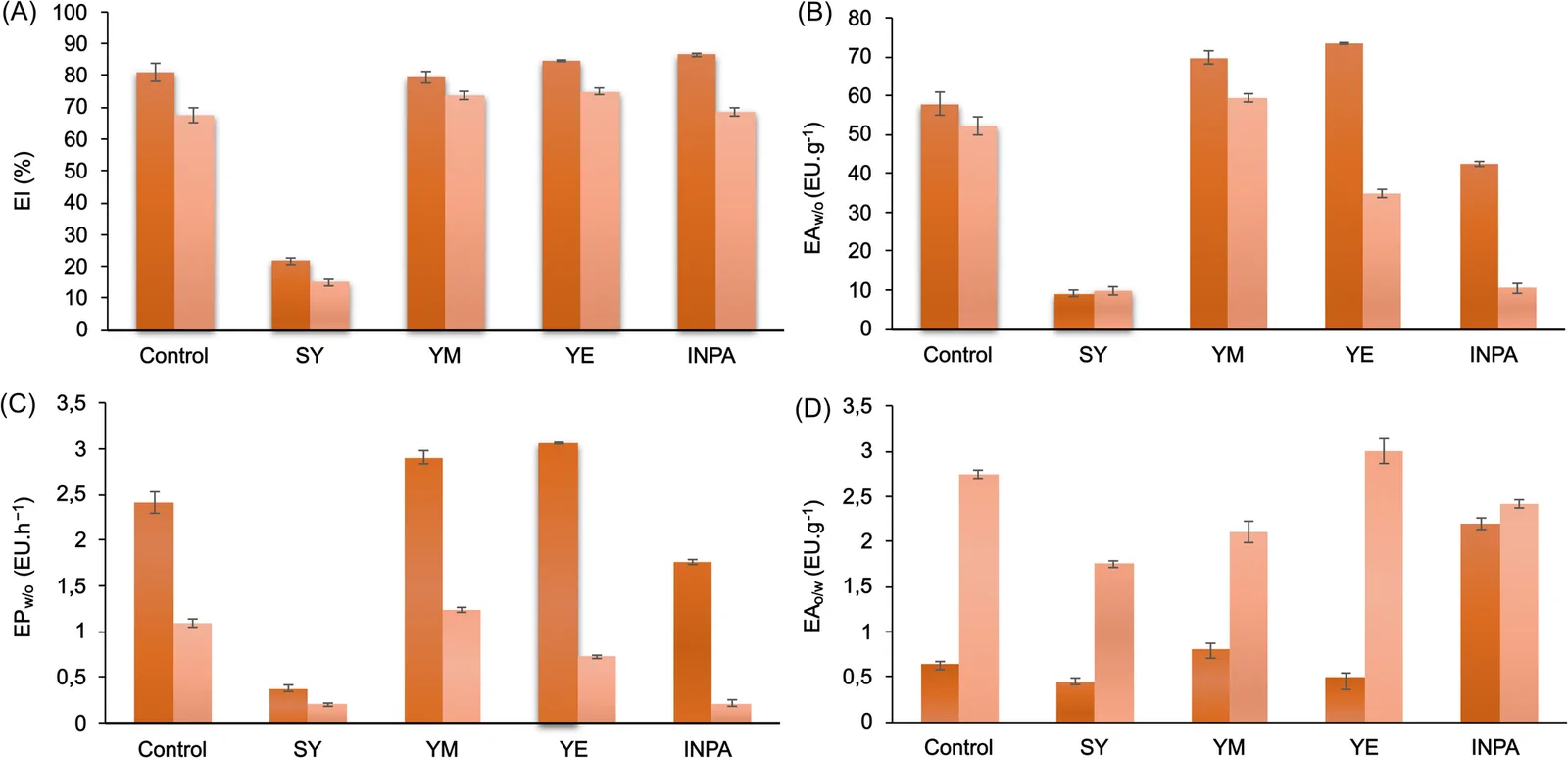
Evaluation of the emulsifying activity of strain INPA R296 in different culture media after 24 h and 48 h of incubation. (A) Emulsification index, EI (%); (B) water-in-oil emulsifying activity (EA_W/O_, EU); (C) time-normalized water-in-oil assay response (EP_W/O_, EU h⁻¹); and (D) oil-in-water absorbance-based response (EA_O/W_, AU). The values were calculated directly from the whole culture broth without mass normalization or dilution and are expressed as assay responses rather than mass-normalized units. The O/W response was determined by absorbance at 610 nm, whereas EI and the W/O response were derived from emulsion-height measurements. Analyses were performed after 24 h and 48 h of cultivation. Values represent mean ± standard deviation from three independent biological replicates

In addition to the differences observed in cell growth, emulsifying activity also varied according to the culture medium and incubation time. The emulsification index (EI%) varied among the evaluated conditions (Fig. 8A). At 24 h, the highest values were observed in INPA (86.44%) and YE (84.70%) media, followed by control (80.89%) and YM (79.52%), whereas SY exhibited the lowest index (21.69%). After 48 h, EI% values decreased under all evaluated conditions, ranging from 15.02% to 74.97%. These temporal differences may reflect changes in medium chemistry, emulsion stability, or the composition of extracellular material [69]. Because the amount of emulsifying material was not quantified, higher EI% values cannot be interpreted as direct evidence of greater compound accumulation or biosurfactant concentration.

In addition to the differences observed in the emulsification index, the O/W and W/O assay responses exhibited distinct profiles during cultivation **(**Fig. 8B-D). Because EA_W/O_-related values were derived from emulsification-index measurements whereas EA_O/W_ was based on absorbance, these parameters are not directly comparable without normalization [70]. We therefore interpret them as complementary assay responses rather than evidence that one emulsion type was intrinsically more efficiently stabilized by a quantified biosurfactant. Differences among media may reflect the combined effects of growth, extracellular components, oil dispersion, and assay chemistry [71].

Consistent with the assay-specific W/O response, the highest EA_W/O_-related values at 24 h were observed in YE (73.54 EU) and YM (69.76 EU) media, followed by control (57.95 EU), whereas SY exhibited the lowest activity (9.08 EU). After 48 h, a reduction in the response was observed in most evaluated conditions, especially in YE and INPA media. The time-normalized values shown as EP_W/O_ were not interpreted as biosurfactant production rates because the amount of biosurfactant was not measured. Accordingly, the results are discussed as emulsifying assay responses rather than quantitative production yields.

Although the W/O-related responses were higher during the first 24 h, the absorbance-based O/W responses remained lower, ranging from 0.45 to 3.00 AU, and increased after 48 h in most media, particularly YE and the control. These patterns may reflect changes in emulsion properties, medium chemistry, or extracellular material and cannot be interpreted as differences in the concentration or accumulation of a specific biosurfactant. Because the O/W and W/O responses are based on different measurements, they should not be directly compared without normalization. For comparison, previous studies reported emulsification responses of similar magnitude for *Bacillus* strains. For example, *B. subtilis* UCP 1533 showed emulsification indices ranging from 50% to 100% depending on the oil tested, including 100% for used motor oil and values between 50% and 100% for vegetable oils [69]. Decesaro et al. [70] reported approximately 59% emulsification of diesel by a *B. pumilus* strain isolated from diesel-contaminated soil. These values cannot be compared directly with the present results because the studies differed in substrate, medium composition, incubation time, use of cell-free or whole broth, purification status, and calculation procedures. Accordingly, the present data are interpreted as operational emulsification responses rather than as a production-yield benchmark.

### Metabolite annotation by GNPS-based molecular networking

**Fig. 9:**
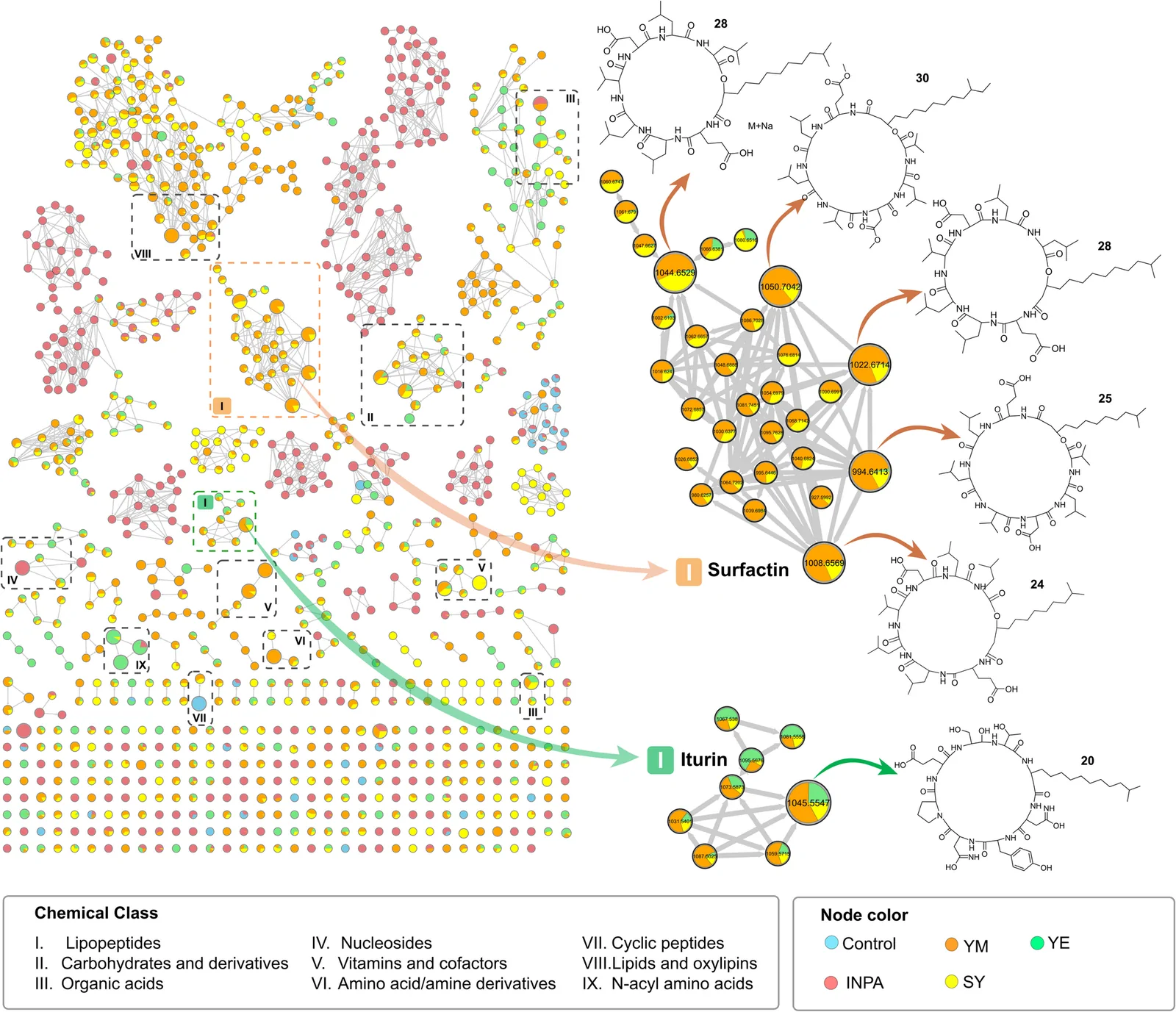
Molecular network obtained on the GNPS2 platform from UHPLC-HRMS/MS data of the crude extract from strain INPA R296. The observed clusters represent different chemical classes putatively annotated by spectral similarity, including lipopeptides, carbohydrates and derivatives, organic acids, nucleosides, vitamins and cofactors, amino acid/amide derivatives, cyclic peptides, lipids and oxylipins, and *N*-acyl amino acids. The expanded details highlight the molecular networks associated with the surfactin (cluster I) and iturin (cluster II) families, containing different putatively annotated congeners. Node size is proportional to the relative abundance of the detected features, whereas colors represent the different culture media supplemented with residual soybean oil

The generation of molecular networks using GNPS enabled the putative annotation of compounds related to primary and specialized metabolism. Annotations were supported by spectral similarity, precursor ion mass accuracy, cosine scores, diagnostic MS/MS fragments, and comparison with literature data. The molecular network and the main chemical families are shown in Fig. 9. All annotations were assigned as MSI level 2.

Different culture media were associated with distinct metabolic profiles, suggesting a medium-dependent variation in the detected features. Surfactin- and iturin-related networks showed higher relative abundance in nutritionally complex media containing yeast extract or other organic components (SY, YM, and YE), consistent with a possible influence of amino acids, vitamins, and metabolic precursors on nonribosomal peptide-associated chemistry [72]. These observations are hypotheses based on metabolite profiles and do not demonstrate a specific nutrient mechanism or BGC expression. A similar pattern was observed for surfactin isoforms (C14, C15, and structural variants), suggesting that medium composition may influence the relative abundance and diversity of detected congeners [73]. Based on *m/z* values, mass errors below ± 5 ppm, and comparison with spectral libraries and literature data, 34 compounds were putatively annotated in the crude extract of strain INPA R296 (Table 3). The metabolite profile included compounds related to central metabolism and amphiphilic lipopeptide chemistry. The annotation of compounds **24**, **26**, **27**, **29** and **31** ([M + Na]⁺) was supported by manual dereplication using exact mass and MS/MS fragmentation patterns consistent with previous reports [74–77]. These compounds were classified as MSI level 2 putative annotations according to Metabolomics Standards Initiative criteria [78, 79].

**Table 3:**
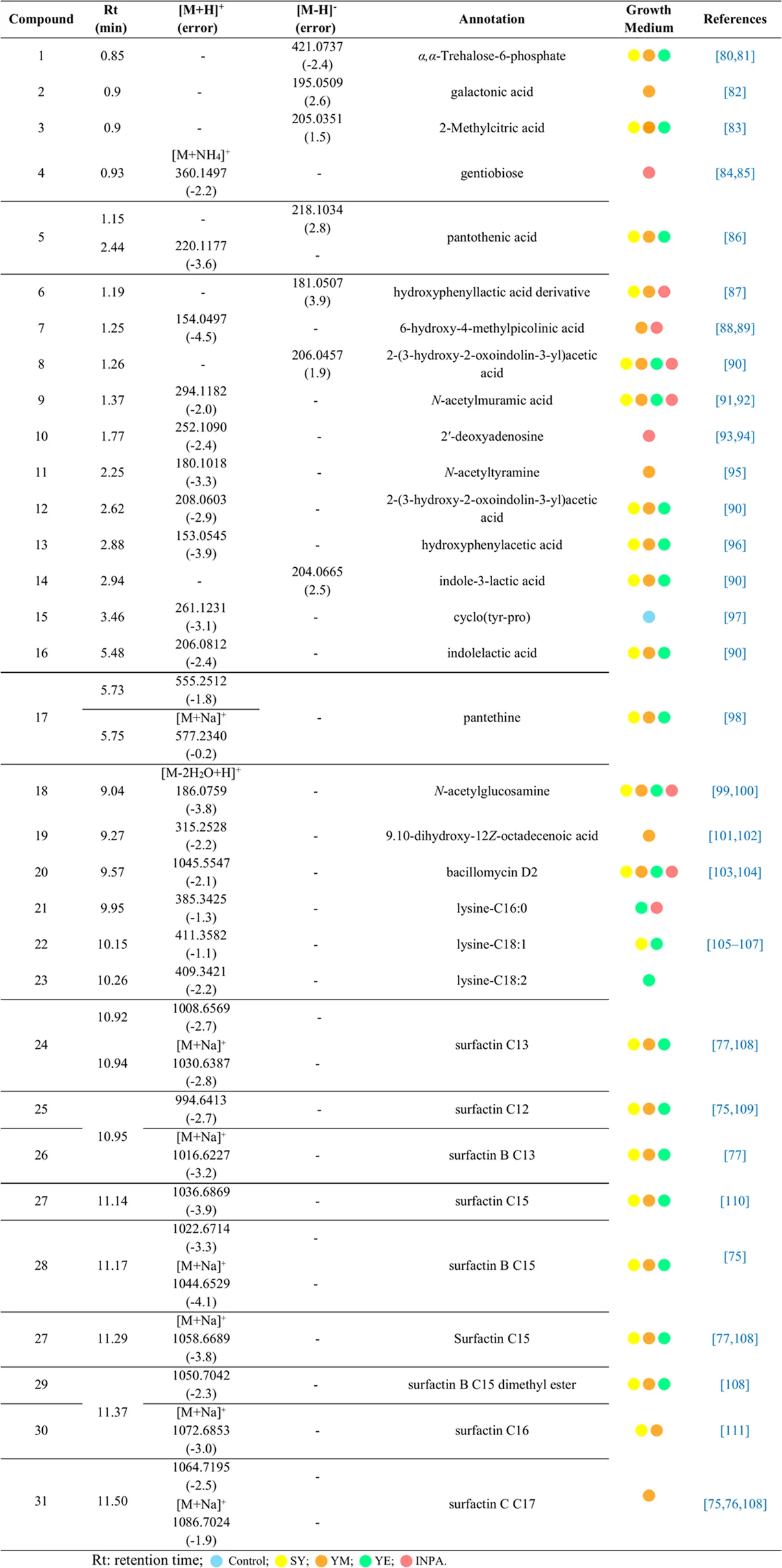
Putatively annotated metabolites in the crude extract of *B. subtilis* subsp. *subtilis* INPA R296. Different adduct forms were observed depending on the chemical properties of the metabolites and the electrospray ionization (ESI) conditions, including protonated species [M+H]⁺, deprotonated species [M−H]⁻, as well as [M+NH4]⁺, [M+Na]⁺, [M+Na+H2O]⁺, and [M−2H2O+H]⁺ adducts. The adduct type is indicated above the *m/z* value when the detected ionic form differs from the expected protonated or deprotonated molecular ion. Values in parentheses correspond to the mass error (ppm) calculated relative to the theoretical mass of the considered adduct. Repeated putative names in different rows correspond to distinct LC-MS features, retention times, or adduct forms and were not interpreted as independent structural confirmations

Among the annotated compounds, pantothenic acid (**5**) and pantethine (**17**), both related to coenzyme A biosynthesis, were identified as metabolites associated with energy metabolism and lipid synthesis [111, 112]. These compounds participate in acyl transfer reactions involved in amphiphilic molecule and lipopeptide biosynthesis [113, 114].

The metabolomic profile also revealed the putative annotation of the hydroxylated fatty acid 9,10-dihydroxy-12*Z*-octadecenoic acid (**19**), an oxylipin commonly associated with emulsifying and antimicrobial properties [115]. In addition, *N*-acyl amino acids, including lysine-C16:0 (**21**), lysine-C18:1 (**22**), and lysine-C18:2 (**23**), were identified. These amphiphilic molecules are associated with antimicrobial and surfactant activities, such as membrane permeabilization and modulation of cell adhesion [116–118].

**Fig. 10:**
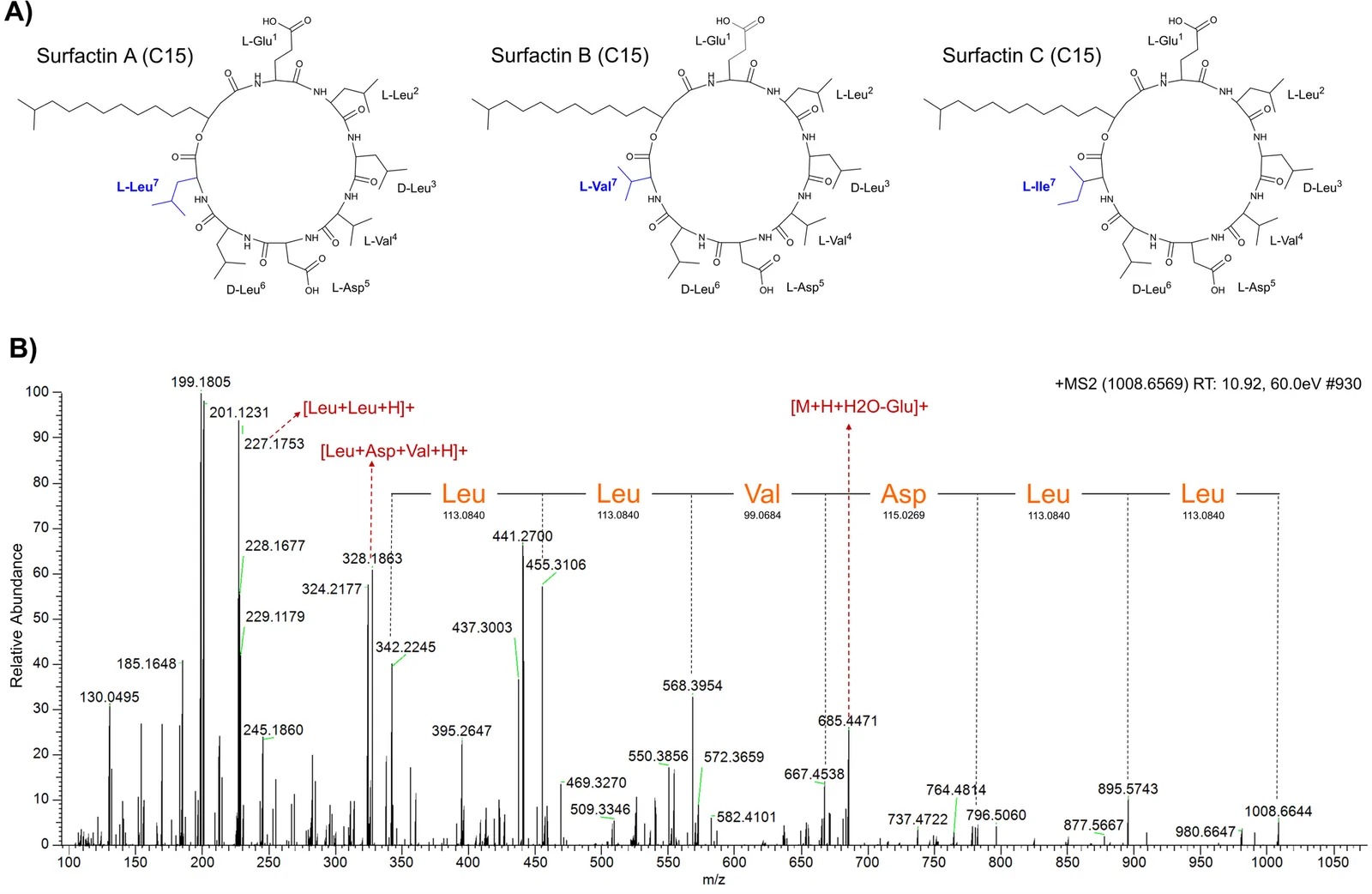
Structural organization and MS/MS fragmentation profile of surfactins detected in the crude extract of *B. subtilis* subsp. *subtilis* INPA R296. (A) Structures of surfactin isoforms, highlighting the amino acid variation at position 7 among surfactin A (Leu_7_), surfactin B (Val_7_), and surfactin C (Ile_7_). (B) Fragmentation spectrum of the precursor ion *m/z* 1008.6569 (Rt = 10.92 min), annotated as surfactin A in the crude extract of strain *B. subtilis* subsp. *subtilis* INPA R296. The MS/MS spectrum obtained in positive ionization mode presented characteristic fragments associated with peptide ring cleavage, including the ions [Leu + Leu+H]⁺, [Leu + Asp+Val + H]⁺, and the neutral loss [M + H+H₂O-Glu]⁺, frequently described for lipopeptides of the surfactin family

Among the putatively annotated compounds, high-molecular-weight cyclic lipopeptides belonging to the surfactin and iturin families stood out, including bacillomycin D2 (**20**), surfactins A, B, and C, and their variants (**24**–**31**). The structural organization of representative surfactin isoforms is shown in Fig. 10A, highlighting amino acid substitutions at position C-7 among surfactin A (Leu7), surfactin B (Val7), and surfactin C (Ile7). These annotations are compatible with cyclic lipopeptides described in the literature as compounds associated with interfacial activity and surface-tension reduction [4, 114], but they were not confirmed with authentic standards or structural isolation. The occurrence of different putative isoforms is also consistent with NRPS biosynthetic systems capable of generating structural diversity through variations in amino acid composition and lipid chain length [11, 119].

The MS/MS fragmentation spectrum of compound **24**, putatively annotated as surfactin A, is presented in Fig. 10B. The precursor ion detected in positive ionization mode at *m/z* 1008.6569 (Rt = 10.92 min) exhibited a mass error of -2.7 ppm and characteristic fragment ions associated with peptide ring cleavage, including [Leu + Leu+H]^+^, [Leu + Asp+Val + H]^+^, and the neutral loss [M + H+H_2_O − Glu]^+^. The fragmentation profile showed high similarity to spectra previously reported for surfactins, supporting its putative annotation with MSI level 2 confidence based on exact mass, MS/MS fragmentation pattern, and comparison with reference spectral data.

The annotation of compound **20**, belonging to the iturin family, is consistent with a putative lipopeptide annotation obtained from the metabolomic data, but no iturin-related BGC was identified in the genome-mining analysis. This discrepancy may reflect limitations in genome annotation, BGC detection, database similarity thresholds, extraction, ionization, or spectral annotation. Conversely, the fengycin-related BGC was predicted but fengycin congeners were not putatively annotated under the conditions evaluated. Possible explanations include lack of expression, the 196 h sampling time, extraction inefficiency, low abundance, ionization limitations, or GNPS filtering criteria. These observations illustrate that genomic prediction and metabolomic annotation provide complementary evidence but do not establish direct BGC–metabolite correspondence. The proposed bacillomycin D2 assignment should therefore be considered MSI level 2 and requires confirmation by authentic standards or targeted structural analysis. The relationship between the predicted BGCs and putatively annotated metabolites is summarized in Table 4.

**Table 4:** Relationship between selected predicted BGCs and putatively annotated metabolites. The table summarizes genomic potential and metabolomic observations without implying direct BGC–metabolite causality

Predicted BGC / region	MIBiG accession	Expected product	Putatively annotated metabolite(s)	Observation	Interpretation
Region 1.1; NRPS, betalactone	BGC0001095	Fengycin	None listed in Table 3	Not detected under the evaluated conditions	Predicted biosynthetic potential; absence may reflect expression, sampling time, extraction, ionization, abundance, or GNPS filtering.
Region 4.1; NRPS	BGC0000433	Surfactin	surfactin C12–C17 and variants	Putatively detected	MSI level 2 annotation; coexistence with the predicted BGC does not establish causality.
Region 2.1; CDPS	BGC0002103	Pulcherriminic acid	None listed in Table 3	Not evaluated as a targeted product	Predicted cluster not linked to a specific detected metabolite.
Region 2.3; other	BGC0001184	Bacilysin			
Region 3.1; transAT-PKS/NRPS	BGC0001089	Bacillaene		Not detected as a targeted annotation	Predicted biosynthetic potential only.
Region 8.1; NRP-metallophore/NRPS	BGC0000309	Bacillibactin			
Iturin-family metabolite	No corresponding iturin BGC identified by the current antiSMASH result	Bacillomycin D2 / iturin-family lipopeptide	bacillomycin D2, *m/z* 1045.5547	Putatively detected	Requires confirmation; the discrepancy may reflect annotation or BGC-detection limitations.

### Potential applications and study limitations

The emulsifying potential observed for strain INPA R296 and its growth in media supplemented with residual soybean oil may be relevant to future studies on bioremediation, agricultural biocontrol, environmental biotechnology, and the valorization of agro-industrial residues. However, these applications were not directly tested in the present work and require validation under application-specific conditions, including stability, toxicity, substrate conversion, and performance in complex environmental or agricultural systems.

For the functional assays, data interpretation was limited to descriptive comparisons of three independent biological replicates per cultivation condition. Because the available dataset was not structured for a full factorial reanalysis across media and time points, no inferential statistical analysis or significance lettering was added.

This study evaluated a single strain and did not quantify biosurfactant yield, surface or interfacial tension, substrate consumption, or gene expression. The metabolite assignments were putative MSI level 2 annotations without authentic-standard confirmation, targeted MS/MS validation, or structural isolation. The different cultivation times used for functional assays and metabolomics also limit direct temporal comparisons, and residual soybean oil may have interfered with OD₆₀₀ measurements. Finally, the data demonstrate coexistence between predicted biosynthetic potential, emulsifying activity, and putative metabolite annotations, but do not establish causal relationships between individual BGCs and metabolites.

## Conclusions

Genomic, metabolomic, and functional analyses of *B. subtilis subsp. subtilis* INPA R296 revealed multiple BGCs associated with specialized metabolite biosynthesis, together with emulsifying activity and putative annotations of *N*-acyl amino acids, oxylipins, and cyclic lipopeptides from the surfactin and iturin families. The genomic results indicate biosynthetic potential, whereas the functional assays demonstrate emulsifying activity under the evaluated conditions.

Culture-medium composition influenced cell growth and emulsifying assay responses, with higher values in some media containing complex organic sources and residual soybean oil. These results identify strain INPA R296 as a candidate for further investigation of amphiphilic metabolites and sustainable use of agro-industrial residues. Direct biosurfactant yield, BGC expression, metabolite structure, and causal relationships between individual BGCs and metabolites remain to be established.

## Supplementary Information

Below is the link to the electronic supplementary material.

Supplementary Material 1 (DOCX 96.9 KB)
