# Supplementary material for: Genomic and functional characterization of Bacillus subtilis subsp. subtilis INPA R296 with emulsifying activity in cultivation with residual soybean oil
Source: Braz J Microbiol. 2026 Oct 9;57(1):288. doi: 10.1007/s42770-026-02098-1 (PMC13653007; doi:10.1007/s42770-026-02098-1)
Supplement: Supplementary file 1 [file 42770_2026_2098_MOESM1_ESM.docx]

**Supplementary material**

**Genomic and functional characterization of *Bacillus subtilis* subsp. *subtilis* INPA R296 producing biosurfactants in cultivation with residual soybean oil**

Suziane P. Rodrigues^1^, José C. I. Silva^4^, Nadionara C. Menezes^1^, Rosineide C. Souza^1^, Aline O. Santos^4^, Igor R. S. Graça^4^, Rogério E. Hanada^1^, Larissa K. Barbosa^2^, Hector H. F. Koolen^4^, Gilvan F. Silva^3^, Luiz A. Oliveira^1^

^1^ Instituto Nacional de Pesquisas da Amazônia (INPA), 69080-971, Manaus, Brazil

^2^ Central de Coleções Microbiológicas (CCM), Universidade do Estado do Amazonas (UEA), 69065-001, Manaus, Brazil

^3^ Embrapa Amazônia Ocidental, 69010-970, Manaus, Brazil

^4^ Grupo de Pesquisas em Metabolômica e Espectrometria de Massas, Universidade do Estado do Amazonas (UEA), 69065-001, Manaus, Brazil

*Corresponding author

Suziane Pinto Rodrigues: [spr.dbb22@uea.edu.br](mailto:spr.dbb22@uea.edu.br)

**Supplementary Fig. S1** Information panel provided by NCBI regarding the complete genome repository of strain INPA R296.

**Supplementary Table S1** Pairwise comparisons between the query genome and the genomes of the closest phylogenomic strains performed on the TYGS platform.

| **Query strain** | **Subject strain** | **dDDH (d0, in %)** | **C.I. (d0, in %)** | **dDDH (d4, in %)** | **C.I. (d4, in %)** | **dDDH (d6, in %)** | **C.I. (d6, in %)** | **G+C content difference (in %)** |
| --- | --- | --- | --- | --- | --- | --- | --- | --- |
| Strain INPA R296 | *Bacillus subtilis* subsp. *Subtilis168* | 90.0 | [86.8 - 92.5] | 88.8 | [86.3 - 90.8] | 92.4 | [90.0 - 94.3] | 0.18 |
|  | *Bacillus stercoris* Mal05 | 84.5 | [80.7 - 87.6] | 62.4 | [59.6 - 65.2] | 83.0 | [79.7 - 85.9] | 0.03 |
|  | *Bacillus spizizenii* TU-B-10 | 81.7 | [77.8 - 85.1] | 50.7 | [48.1 - 53.4] | 77.3 | [73.8 - 80.4] | 0.13 |
|  | *Bacillus inaquosorum* A65.1 | 79.4 | [75.4 - 82.9] | 50.4 | [47.8 - 53.0] | 75.2 | [71.8 - 78.4] | 0.34 |
|  | *Bacillus rugosus* A78.1 | 83.8 | [80.0 - 87.0] | 48.8 | [46.2 - 51.4] | 78.2 | [74.8 - 81.3] | 0.17 |
|  | *Bacillus cabrialesii* TE3 | 84.3 | [80.5 - 87.4] | 47.6 | [45.0 - 50.2] | 78.2 | [74.8 - 81.3] | 0.53 |
|  | *Bacillus tequilensis EA-CB0015* | 74.6 | [70.6 - 78.2] | 44.9 | [42.4 - 47.5] | 69.3 | [65.9 - 72.5] | 0.05 |
|  | *Bacillus vallismortis*  Bac111 | 75.4 | [71.4 - 79.0] | 42.8 | [40.3 - 45.4] | 69.1 | [65.7 - 72.3] | 0.46 |
|  | *Bacillus mexicanus*  MoR6 | 55.7 | [52.1 - 59.1] | 39.7 | [37.2 - 42.2] | 52.2 | [49.1 - 55.3] | 2.62 |
|  | *Bacillus halotolerans* ZB201702 | 81.5 | [77.6 - 84.9] | 32.7 | [30.3 - 35.2] | 68.2 | [64.8 - 71.4] | 0.12 |
|  | *Bacillus mojavensis* UCMB5075 | 80.8 | [76.8 - 84.1] | 32.6 | [30.2 - 35.1] | 67.5 | [64.1 - 70.8] | 0.09 |
|  | *Bacillus gibsonii* DSM 8722 | 12.6 | [9.9 - 15.9] | 26.3 | [23.9 - 28.7] | 13.0 | [10.7 - 15.7] | 3.3 |
|  | *Bacillus atrophaeus* SW | 45.8 | [42.5 - 49.3] | 22.1 | [19.8 - 24.5] | 37.5 | [34.5 - 40.5] | 0.52 |
|  | *Bacillus siamensis* M54 | 31.8 | [28.4 - 35.3] | 20.7 | [18.4 - 23.1] | 27.9 | [24.9 - 31.0] | 2.84 |
|  | *Bacillus amyloliquefaciens* GKT04 | 31.7 | [28.3 - 35.3] | 20.6 | [18.3 - 23.0] | 27.8 | [24.9 - 30.9] | 2.59 |
|  | *Bacillus nakamurai* NRRL B-41091 | 32.9 | [29.5 - 36.4] | 20.5 | [18.2 - 22.9] | 28.5 | [25.6 - 31.6] | 1.57 |
|  | *Jeotgalibacillus marinus*  DSM 1297 | 12.8 | [10.1 - 16.1] | 19.4 | [17.2 - 21.8] | 13.2 | [10.8 - 15.9] | 4.81 |
|  | *Bacillus licheniformis* ATCC 14580 | 16.7 | [13.7 - 20.2] | 18.7 | [16.5 - 21.1] | 16.4 | [13.9 - 19.4] | 2.52 |
|  | *Micromonospora provocatoris* B-183 | 12.6 | [9.9 - 15.9] | 18.6 | [16.4 - 21.0] | 13.0 | [10.7 - 15.7] | 6.34 |

**Supplementary Table S2** Strains used in the TYGS platform and their respective datasets.

| **TYGS ID** | **Preferred name** | **Deposit** | **Base pairs** | **Percent G+C** | **No. proteins** | **Bioproject accession** | **Biosample accession** | **Assembly accession** |
| --- | --- | --- | --- | --- | --- | --- | --- | --- |
| U1168285 | *Bacillus subtilis* subsp. *Subtilis* | INPA R296 | 4.270.493 | 43,69 | 4352 | PRJNA1427619 | SAMN55925958 |  |
| U1168286 | *Bacillus subtilis* subsp. *Subtilis* | 168 | 4.215.606 | 43,51 | 4226 | PRJNA76 | SAMEA3138188 | GCA 000009045.1 |
| U1168287 | *Bacillus spizizenii* | TU-B-10 | 4.207.222 | 43,82 | 4149 | PRJNA68561 | SAMN02603352 | GCA 000227465.1 |
| U1168288 | *Bacillus nakamurai* | NRRL B-41091 | 3.689.277 | 45,26 | 3827 | PRJNA299292 | SAMN04450409 | GCA 001584325.1 |
| U1168289 | *Bacillus vallismortis* | Bac111 | 3.958.372 | 44,15 | 3921 | PRJNA412146 | SAMN10146793 | GCA 003667885.1 |
| U1168290 | *Bacillus halotolerans* | ZB201702 | 4.154.245 | 43,81 | 4130 | PRJNA470606 | SAMN09095924 | GCA 004006435.1 |
| U1168291 | *Bacillus cabrialesii* | TE3 | 4.125.766 | 44,22 | 4062 | PRJNA504313 | SAMN10390288 | GCA 004124315.2 |
| U1168292 | *Bacillus tequilensis* | EA-CB0015 | 4.012.371 | 43,74 | 4085 | PRJNA606711 | SAMN14111977 | GCA 012225885.1 |
| U1168293 | *Bacillus mojavensis* | UCMB5075 | 4.031.121 | 43,78 | 4045 | PRJNA616328 | SAMN14479759 | GCA 012648005.1 |
| U1168294 | *Bacillus amyloliquefaciens* | GKT04 | 4.149.690 | 46,28 | 4064 | PRJNA716416 | SAMN18435563 | GCA 019396925.1 |
| U1168295 | *Bacillus rugosus* | A78.1 | 4.146.938 | 43,86 | 4037 | PRJNA831700 | SAMN27761610 | GCA 023238245.1 |
| U1168296 | *Bacillus inaquosorum* | A65.1 | 4.242.130 | 44,03 | 4131 | PRJNA831700 | SAMN27761609 | GCA 023238325.1 |
| U1168297 | *Bacillus gibsonii* | DSM 8722 | 3.932.415 | 40,39 | 4039 | PRJNA931000 | SAMN33041223 | GCA 028610635.1 |
| U1168298 | *Bacillus stercoris* | Mal05 | 4.282.228 | 43,66 | 4271 | PRJNA952856 | SAMN34090420 | GCA 029772985.1 |
| U1168299 | *Bacillus licheniformis* | ATCC 14580 | 4.227.253 | 46,21 | 4291 | PRJNA1037563 | SAMN38186259 | GCA 034478925.1 |
| U1168300 | *Bacillus atrophaeus* | SW | 4.292.910 | 43,17 | 4168 | PRJNA1104434 | SAMN41078598 | GCA 039519175.1 |
| U1168301 | *Jeotgalibacillus marinus* | DSM 1297 | 3.521.308 | 38,88 | 3620 | PRJNA1134393 | SAMN42419938 | GCA 040801915.1 |
| U1168302 | *Bacillus mexicanus* | MoR6 | 4.299.957 | 41,07 | 4543 | PRJNA1095867 | SAMN40738003 | GCA 050445615.1 |
| U1168303 | *Micromonospora provocatoris* | B-183 | 1.117.909 | 37,35 | 1103 | PRJNA802201 | SAMN41009153 | GCA 050465265.1 |
| U1168304 | *Bacillus siamensis* | M54 | 3.897.476 | 46,53 | 3747 | PRJNA1108901 | SAMN41257608 | GCA 051628075.1 |

**Supplementary Table S3** Organisms used for phylogenomic tree construction by AutoMLST, with emphasis on estimated ANI values.

| **Query organism** | **Reference assembly ID** | **Reference name** | **Mash distance** | **estimated ANI** | **Genus** | **Order** | **Type strain** |
| --- | --- | --- | --- | --- | --- | --- | --- |
| Strain INPA R296 | GCF_000155325 | *Bacillus subtilis* subsp. *subtilis* str. 168 | 0.0139 | 98.6% | Bacillus | Bacillales | True |
|  | GCF_000186085 | *Bacillus subtilis* subsp. *subtilis* str. NCIB 3610 | 0.0140 | 98.6% | Bacillus | Bacillales | True |
|  | GCF_002027305 | *Bacillus intestinalis* | 0.0628 | 93.7% | Bacillus | Bacillales | True |
|  | GCF_001274925 | *Jeotgalibacillus marinus* | 0.0631 | 93.7% | Jeotgalibacillus | Bacillales | True |
|  | GCF_000507145 | *Bacillus tequilensis* KCTC 13622 | 0.0785 | 92.2% | Bacillus | Bacillales | True |
|  | GCF_000245315 | *Bacillus vallismortis* DV1-F-3 | 0.0885 | 91.1% | Bacillus | Bacillales | True |
|  | GCF_001517105 | *Bacillus halotolerans* | 0.1079 | 89.2% | Bacillus | Bacillales | True |
|  | GCF_001637525 | *Bacillus halotolerans* | 0.1079 | 89.2% | Bacillus | Bacillales | True |
|  | GCF_000507105 | *Bacillus mojavensis* | 0.1115 | 88.8% | Bacillus | Bacillales | True |
|  | GCF_000245335 | *Bacillus mojavensis* | 0.1116 | 88.8% | Bacillus | Bacillales | True |
|  | GCF_001584335 | *Bacillus atrophaeus* | 0.1680 | 83.2% | Bacillus | Bacillales | True |
|  | GCF_000262045 | *Bacillus siamensis* KCTC 13613 | 0.1810 | 81.9% | Bacillus | Bacillales | True |
|  | GCF_000966575 | *Bacillus siamensis* | 0.1818 | 81.8% | Bacillus | Bacillales | True |
|  | GCF_000196735 | *Bacillus amyloliquefaciens* DSM 7 | 0.1849 | 81.5% | Bacillus | Bacillales | True |
|  | GCF_001584325 | *Bacillus nakamurai* | 0.1868 | 81.3% | Bacillus | Bacillales | True |
|  | GCF_001307105 | *Bacillus australimaris* | 0.2338 | 76.6% | Bacillus | Bacillales | True |
|  | GCF_001592005 | *Bacillus sonorensis* NBRC 101234 = KCTC 13918 | 0.2351 | 76.5% | Bacillus | Bacillales | True |
|  | GCF_000008425 | *Bacillus licheniformis* | 0.2378 | 76.2% | Bacillus | Bacillales | True |
|  | GCF_000011645 | *Bacillus licheniformis* | 0.2378 | 76.2% | Bacillus | Bacillales | True |
|  | GCF_000300535 | *Bacillus xiamenensis* | 0.2392 | 76.1% | Bacillus | Bacillales | True |
|  | GCF_000691145 | *Bacillus altitudinis* 41KF2b | 0.2407 | 75.9% | Bacillus | Bacillales | True |
|  | GCF_001457015 | *Bacillus cellulasensis* | 0.2422 | 75.8% | Bacillus | Bacillales | True |
|  | GCF_000691165 | *Bacillus safensis* FO-36b | 0.2470 | 75.3% | Bacillus | Bacillales | True |
|  | GCF_000715205 | *Bacillus zhangzhouensis* | 0.2470 | 75.3% | Bacillus | Bacillales | True |
|  | GCF_001278705 | *Bacillus gobiensis* | 0.2736 | 72.6% | Bacillus | Bacillales | True |

**Supplementary Table S4** Targeted functional annotation of genes located in the NRPS-type biosynthetic cluster (Region 1.1) identified by antiSMASH in the genome of strain INPA R296. Gene functions were assigned based on the functional annotation provided by the BV-BRC (PATRIC) platform, considering genomic coordinates, protein families (PGFAM), and functional classification within the cluster.

| **Gene (antiSMASH)** | **Cluster role (antiSMASH)** | **Contig (PATRIC)** | **Genomic coordinates (bp)** | **Functional annotation (PATRIC)** | **Protein family (PGFAM)** |
| --- | --- | --- | --- | --- | --- |
| g52 | Biosynthetic-additional | contig_1 | 72,921–73,814 | UTP–glucose-1-phosphate uridylyltransferase | PGF_00064393 |
| g55 | Biosynthetic-additional | contig_1 | 75,901–77,430 | Methylcrotonyl-CoA carboxylase, carboxyl transferase subunit | PGF_05369673 |
| g56 | Biosynthetic-additional | contig_1 | 77,447–78,229 | Methylglutaconyl-CoA hydratase | PGF_00943546 |
| g57 | Biosynthetic | contig_1 | 78,250–79,149 | Hydroxymethylglutaryl-CoA lyase | PGF_02980852 |
| g59 | Biosynthetic-additional | contig_1 | 79,400–80,734 | Biotin carboxylase | PGF_00404321 |
| g60 | Biosynthetic | contig_1 | 80,744–82,393 | Long-chain fatty-acid–CoA ligase | PGF_00017580 |
| g61 | Biosynthetic-additional | contig_1 | 82,437–83,579 | Isovaleryl-CoA dehydrogenase | PGF_00032869 |
| g64 | Biosynthetic | contig_1 | 86,409–90,182 | Polyketide synthase | PGF_10503453 |
| g65 | Biosynthetic | contig_1 | 90,256–101,055 | Polyketide synthase | PGF_10503453 |
| g66 | Biosynthetic | contig_1 | 101,081–108,610 | Polyketide synthase | PGF_10503453 |
| g67 | Biosynthetic | contig_1 | 108,767–116,458 | Polyketide synthase | PGF_10503453 |
| g68 | Biosynthetic | contig_1 | 116,474–124,159 | Polyketide synthase | PGF_10503453 |
| g71 | Transport | contig_1 | 127,161–128,552 | Na⁺-driven multidrug efflux pump | PGF_05837706 |
| g74 | Biosynthetic-additional | contig_1 | 130,876–132,639 | Gamma-glutamyltransferase | PGF_02890229 |
| g75 | Biosynthetic-additional | contig_1 | 133,787–134,776 | Alcohol dehydrogenase | PGF_03572345 |
| g76 | Biosynthetic-additional | contig_1 | 134,833–136,314 | Glutamate synthase | PGF_10440725 |
| g78 | Regulatory | contig_1 | 141,040–141,942 | Glutamate synthase regulatory protein | PGF_00007795 |

**Supplementary Table S5** Targeted functional annotation of genes located in the NRPS-type biosynthetic cluster (Region 4.1) identified by antiSMASH in the genome of strain INPA R296. Gene functions were assigned based on the functional annotation provided by the BV-BRC (PATRIC) platform, considering genomic coordinates, protein families (PGFAM), and functional classification within the cluster.

| **Gene (antiSMASH)** | **Cluster role (antiSMASH)** | **Contig (PATRIC)** | **Genomic coordinates (bp)** | **Functional annotation (PATRIC)** | **Protein family (PGFAM)** |
| --- | --- | --- | --- | --- | --- |
| g2319 | Transport | contig_4 | 193,293–194,771 | Di-/tripeptide/cation symporter (DtpT) | PGF_02030730 |
| g2322 | Biosynthetic-additional | contig_4 | 197,852–198,430 | Hydroxyaromatic non-oxidative decarboxylase | PGF_03299849 |
| g2323 | Regulatory | contig_4 | 198,557–199,429 | LysR-family transcriptional regulator (BsdA) | PGF_00017893 |
| g2324 | Biosynthetic-additional | contig_4 | 199,545–200,351 | L-cystine ABC transporter, substrate-binding protein | PGF_00912542 |
| g2325 | Transport | contig_4 | 200,338–201,042 | L-cystine ABC transporter permease protein (TcyB) | PGF_07474537 |
| g2326 | Transport | contig_4 | 201,056–201,799 | ABC transporter ATP-binding protein | PGF_08789310 |
| g2328 | Biosynthetic-additional | contig_4 | 202,873–203,547 | 4′-phosphopantetheinyl transferase | PGF_10369163 |
| g2329 | Biosynthetic-additional | contig_4 | 203,542–204,876 | Transcriptional regulator, GntR family | PGF_09985786 |
| g2330 | Transport | contig_4 | 205,000–205,938 | Uncharacterized transporter, EamA family | PGF_02907590 |
| g2331 | Transport | contig_4 | 205,950–206,780 | Uncharacterized membrane protein | PGF_XXXXX |
| g2332 | Biosynthetic-additional | contig_4 | 207,891–208,619 | Thioesterase | PGF_04367281 |
| g2333 | Biosynthetic (core) | contig_4 | 208,648–212,475 | Nonribosomal peptide synthetase module | PGF_10503453 |
| g2334 | Biosynthetic (core) | contig_4 | 212,512–223,260 | Nonribosomal peptide synthetase module | PGF_10503453 |
| g2335 | Biosynthetic (core) | contig_4 | 223,273–234,036 | Nonribosomal peptide synthetase module | PGF_10503453 |
| g2336 | Regulatory | contig_4 | 234,610–234,972 | Transcriptional regulator, HxlR family | PGF_10420765 |
| g2343 | Regulatory | contig_4 | 241,892–242,314 | Transcriptional regulator, MarR family | PGF_00797655 |
| g2346 | Biosynthetic-additional | contig_4 | 243,688–244,551 | ABC transporter substrate-binding protein | PGF_01190544 |
| g2347 | Transport | contig_4 | 244,561–245,241 | ABC transporter permease protein | PGF_00051938 |
| g2348 | Biosynthetic-additional | contig_4 | 245,290–246,483 | Zinc-binding GTPase (YciC) | PGF_00069882 |
| g2351 | Transport | contig_4 | 248,527–249,732 | Nitrate transporter (NasA) | PGF_06014884 |
| g2352 | Biosynthetic-additional | contig_4 | 249,912–252,227 | Assimilatory nitrate reductase electron transfer protein | PGF_02890512 |
